# Need for Affiliation as a Motivational Add-On for Leadership Behaviors and Managerial Success

**DOI:** 10.3389/fpsyg.2016.01972

**Published:** 2016-12-22

**Authors:** Barbara Steinmann, Sonja K. Ötting, Günter W. Maier

**Affiliations:** Work and Organizational Psychology, Department of Psychology, Bielefeld UniversityBielefeld, Germany

**Keywords:** implicit motives, need for affiliation, interactive effects, leadership behaviors, leadership success

## Abstract

In a sample of 70 leader-follower dyads, this study examines the separate and interactive effects of the leaders’ implicit needs for power, achievement, and affiliation on leadership behaviors and outcomes. Results show that whereas the need for achievement was marginally associated with follower-rated passive leadership, the need for affiliation was significantly related to ratings of the leaders’ concern for the needs of their followers. Analyzing motive combinations in terms of interactive effects and accounting for the growing evidence on the value of affiliative concerns in leadership, we assumed the need for affiliation would channel the interplay among the needs for power and achievement in such a way that the leaders would become more effective in leading others. As expected, based on high need for achievement, the followers were more satisfied with their jobs and with their leaders and perceived more transformational leadership behavior if power-motivated leaders equally had a high need for affiliation. Moreover, the leaders indicated higher career success when this was the case. However, in indicators of followers’ performance, the three-way interaction among the needs for power, achievement, and affiliation did not account for additional variance.

## Introduction

In the field of leadership research, a motivational approach has had a long tradition. Apart from studying explicit motivational constructs such as the leader’s motivation to lead (Chan and Drasgow, 2001), scholars have been equally interested in the impact that implicit motivational processes exert on leaders’ effectiveness. The growing body of research in this field acknowledges that leaders’ implicit motives play an important role in corporate and political leadership (Winter, 2010). Early studies (e.g., McClelland and Boyatzis, 1982) found that effective leaders exhibited high levels of the need for power (*n*Pow) and activity inhibition (AI), but low levels of the need for affiliation (*n*Aff). However, evidence on the value of *n*Aff to leadership success has been steadily increasing over time (e.g., Kirkpatrick et al., 2002; Steinmann et al., 2015).

Over the years, studies on leaders’ motive dispositions have not only revealed changes concerning the value of certain implicit motives, but have also accounted for methodological refinements. Research has generally assumed that implicit motives simultaneously influence a person’s behavior (McClelland, 1992). To analyze this combined impact, scholars have usually clustered these motives into dichotomous typological patterns based on pre-defined cut-off values instead of considering more statistically sound linear additive combinations (McClelland, 1992). Resuming work by Spangler and House (1991), Steinmann et al. (2015) have only recently taken a dimensional approach in analyzing the combined impact of motives on successful leadership. Not only does a dimensional approach determine the extent to which each motive contributes to a certain outcome, but it also sheds light on the extent of their interplay. In their study, the authors found that *n*Pow, AI, and *n*Aff actually interact and moderate one another in predicting leaders’ performance.

In the present study, we merge these streams of research and integrate findings on the value of *n*Aff for leadership success with those on the interaction between motives. In doing so, we consider the entirety of the motivational Big Three, that is the needs for power, achievement (*n*Ach), and affiliation. We suggest that in current work environments—where competitors are abundant, innovation cycles are shortened, and products as well as production systems are constantly altered by the ongoing digitization—a high *n*Pow is not enough for leaders to be effective. In order to channel the impact of *n*Pow, a distinct *n*Ach is needed to align their leadership behaviors with the avoidance of routines, the achievement of unique accomplishments, and a drive to excel in competitive situations. Taking into account the evidence on its importance, we suggest that the need for affiliation channels this interaction such that it leads to more effective leadership. This assumption is based on the fact that the sharing of responsibilities, the coaching and development of others, and relational skills are crucial in today’s economy (Post, 2015). More precisely, we assume that contingent on high *n*Ach, *n*Pow more closely relates to the followers’ satisfaction and performance as well as the leaders’ career success if high compared to low *n*Aff adds to this interplay. Moreover, we assume that implicit motives and the interplay between them will exhibit themselves in certain leadership behaviors. Whereas *n*Ach relates to perceptions of passive leadership and *n*Aff to those of a leader’s concern for her or his followers’ needs, *n*Pow that is channeled by high *n*Ach more closely relates to follower-rated transformational leadership if further modulated by high *n*Aff.

In studying these assumptions, we aim to contribute to the field of implicit motives and leadership success in three ways. First, we further illuminate the interplay among the needs for power, achievement, and affiliation, and examine whether these motives modulate each other. This specifically contributes to theory building in the field of implicit motives because, until now, the expression of implicit motives has only been assumed to be channeled by one’s AI and explicit personality constructs (e.g., McClelland et al., 1972, 1989). Second, we assume high *n*Aff benefits leadership success. In doing so, we help to clarify and strengthen the value of the concern for establishing, maintaining, and restoring positive relationships (Heyns et al., 1958) in the leadership context and update theories on implicit motives and leadership which date back more than 40 years. Third, we link implicit motives to leadership behaviors. We empirically verify theoretically derived motive-behavior relationships which have not been examined yet; moreover, we aim to show that perceptions of leadership behavior vary depending on the interplay of motives. Thus, we contribute to furthering knowledge on the behavioral manifestation of implicit motives in the leadership arena.

### The Association of Implicit Motives and Leadership Success

Implicit motives are subconscious affective preferences for certain types of incentives a person perceives as pleasurable (Schultheiss et al., 2008) that develop during childhood (McClelland and Pilon, 1983). These motives are represented non-linguistically (Brunstein, 2008), which is why they may not be intentionally verbalized. Thus, projective measures are needed to assess a person’s motive disposition (Hofer et al., 2010). Despite their conscious inaccessibility, implicit motives determine how one feels and behaves (Fodor, 2010). As they orient, select, and energize spontaneous long-term behavioral trends (McClelland, 1987), implicit motives may be conceived of as general dispositions to act in a specific way (Hofer and Busch, 2011). Research has mainly concentrated on three major motives: the needs for power, achievement, and affiliation. People motivated by *n*Pow derive pleasure from influencing the behavior, emotions, and beliefs of others or the world at large; they strive to control and impress those around them, and are concerned with fame and reputation (Winter, 1973, 1994). Individuals, who are motivated by *n*Ach value performance, seek to improve their skills, and strive for excellence and unique accomplishments (McClelland et al., 1953; Brunstein and Maier, 2005). Those driven by *n*Aff wish to establish, maintain, and restore close and friendly relationships (Heyns et al., 1958). They seek pleasure from affiliate activities and feel sad if separated from others (Winter, 1994). Although *n*Pow, *n*Ach, and *n*Aff find expression in certain behaviors, the link between the arousal of these motives and their behavioral manifestation is not straightforward. The way motives are acted out is modulated by the degree of activity inhibition inherent in a person. AI is not an implicit motive itself, but a stable tendency refining the manner in which motives become manifest (Schultheiss et al., 2009). If people are high in AI, they are able to inhibit the expression of emotional and motivational impulses (McClelland, 1979). For a long time, AI has solely been regarded in relation to *n*Pow. Spangler et al. (2014) have recently extended this concept by postulating that AI equally modulates the behavioral expression of a leader’s *n*Ach and *n*Aff.

Implicit motives and AI have been linked to a wide range of human behaviors and long-term outcomes such as creative performance, task choice, reasoning abilities, decision-making, risk-taking, physical and psychological well-being, and career success. Many studies have linked motives to effective leadership, a field essentially shaped by McClelland and Boyatzis’ (1982) pioneering work. Following McClelland’s (1975) theoretical work, these scholars assumed that leaders are particularly effective if they are high in *n*Pow and AI, but low in *n*Aff. Based on pre-defined cut-off values for both motives and AI, leaders were divided into those showing the respective motive configuration—the so-called Leadership Motive Pattern (LMP)—and those who exhibited a different pattern of implicit motives. Their longitudinal study showed that, after eight and 16 years, the LMP-leaders did indeed attain significantly higher levels of promotion than those who deviated from this pattern.

#### The Role of Need for Power for Successful Leadership

A highly pronounced *n*Pow is a vital leadership characteristic as it inherently motivates people to seek pleasure from influencing others (e.g., McClelland and Boyatzis, 1982). Leadership positions further provide the reputation and status power-motivated people strive for and offer opportunities to control or regulate subordinates (see Spangler and House, 1991). Thus, they satisfy the needs of those high in *n*Pow in several ways. As such positions are particularly satisfying for these persons they sustain their interest in leading on the long run (House and Aditya, 1997). However, *n*Pow has also been related to aggressive, antisocial, and (morally) reprehensible conduct (Winter, 1973, 2000)—behaviors which contradict good leadership practice. Whether power is used in a socially responsible or self-serving fashion depends on the leader’s AI. To be an effective leader, high AI is needed to channel the leader’s *n*Pow. Whereas power-motivated leaders with low AI influence others so as to benefit and aggrandize themselves, those with high AI use their influence for the benefit of others (Magee and Langner, 2008). In corporate contexts, the latter use their impact to attain organizational goals, attempt to maintain the organization’s system, and duly stick to procedures (McClelland and Boyatzis, 1982; Spangler and House, 1991).

The contribution of *n*Pow to leadership performance has been evidenced by a multitude of studies (e.g., McClelland and Boyatzis, 1982; Spangler and House, 1991; Jacobs and McClelland, 1994). Some researchers, though, report a negative relation to indicators of leadership effectiveness, or no relation at all (House et al., 1991; De Hoogh et al., 2005; Delbecq et al., 2013). This negative association is hardly surprising if a self-serving use of power is taken into account. Studies on the socialized use of power have mostly condensed it with AI and *n*Aff and have not examined it separately from the LMP (e.g., McClelland and Boyatzis, 1982), or operationalized responsible power irrespective of AI (Winter, 1991). The few studies that have indeed examined the interactive effect of *n*Pow and AI have either failed to provide evidence on its contribution to leadership performance (Spangler and House, 1991) or found its value to be dependent on the organizational context (De Hoogh et al., 2005). On empirical grounds, the moderating effect theoretically ascribed to AI has thus not been unequivocally supported.

#### The Role of Need for Achievement for Successful Leadership

In their seminal work, McClelland and Boyatzis (1982) found that a leader’s *n*Ach only contributes to leadership success or promotion in lower or technical management positions. In these jobs, advancements depend on professional contributions and not one’s ability to lead others, a skill needed in higher management (McClelland and Boyatzis, 1982). In their studies, De Hoogh et al. (2005) as well as Delbecq et al. (2013) failed to show that the leaders’ *n*Ach relates to their followers’ motivation, work attitudes, performance, or teamwork. In politics, significant or marginal relations have been found between a president’s *n*Ach and his action, perceived greatness, social, or international relations performance (House et al., 1991; Spangler and House, 1991). These associations were negative, though. As leaders that are solely motivated by *n*Ach are concerned with attaining goals through their own efforts rather than those of others (Spangler et al., 2014), they are reluctant to delegate responsibilities (House and Aditya, 1997) and try to keep control over all aspects of their job (McClelland and Burnham, 1976). They avoid making decisions and are not interested in enforcing authority (Spangler et al., 2014). Thus, leaders high in *n*Ach share fundamental characteristics with those who lead passively: Passive leaders also avoid making decisions, abdicate from their leadership responsibilities, and do not take any regulative actions until problems become so severe that groups run the risk of not finishing their tasks (Bass, 1990). Therefore, we assume:

*Hypothesis 1:* A leader’s *n*Ach positively relates to perceptions of passive leadership.

As it stimulates one’s concern for achieving things personally, some suggest that a highly developed *n*Ach benefits success in small-scale businesses, sales, or entrepreneurial positions (McClelland, 1977). The positive association between *n*Ach and entrepreneurial behavior has found meta-analytic support (Collins et al., 2004). Those characterized by high *n*Ach energetically engage in actions, derive pleasure from being responsible for tasks, and devote themselves to innovative activities that involve planning the future (McClelland et al., 1958)—all of which are behaviors and attributes that are vital for successful entrepreneurs. Schumpeter (1934) argued that the key distinction between entrepreneurs and conventional managers is their focus on innovation. In today’s work environments, innovativeness has turned into a critical success factor of corporate performance, wealth creation, and long-term survival (Anderson et al., 2014). Innovations directly relate to an organization’s brand performance (Weerawardena et al., 2006) and clearly increase its stock returns (Srinivasan et al., 2009), which shows that innovations enhance an organization’s competitive advantage (Anderson et al., 2014). Although innovative employee behavior was once regarded as being inappropriate or subversive (Anderson et al., 2004), innovativeness and creativity are now essential demands imposed on employees (Rajan and Wulf, 2006). People high in *n*Ach strive for exceptional accomplishments (Winter, 1994), avoid routines, and try to improve things (McClelland, 1985). Their motivational make-up therefore fosters their involvement in corporate innovations.

In his definition, McClelland (1961) referred to entrepreneurs as leaders with profound decision-making responsibilities. As decision-making entails certain risks, it should be particularly satisfying for people high in *n*Ach because they derive pleasure from engaging in moderately risky tasks (Collins et al., 2004). In contemporary organizations, considerable decision-making authority has been delegated from top to lower management levels (Rajan and Wulf, 2006) so that leaders are given enormous decision-making responsibilities. Compared to the time when research on the link between motives and leadership emerged, leadership today requires much more entrepreneurial behaviors, thus underscoring the fact that *n*Ach is a vital motivational disposition for conventional leaders.

#### The Role of Need for Affiliation for Successful Leadership

A highly developed implicit need for affiliation has been assumed to be detrimental to leadership success; and research has lent support to this assumption (McClelland and Boyatzis, 1982; House et al., 1991; Spangler and House, 1991; Jacobs and McClelland, 1994). It has been reasoned that the need to initiate and maintain positive relations with others runs counter to necessary leadership tasks. Affiliative leaders are reluctant to monitor their followers’ performance, to give negative feedback, or to impose sanctions on them (McClelland, 1985). As they focus on personal relationships, they base their decisions on affection instead of corporate necessities and overly worry about being liked by their followers (McClelland and Boyatzis, 1982; Spangler and House, 1991). People with high *n*Aff try to avoid conflicts, and—as they tend to do everything to sustain the good will of others—make exceptions to the rules based on the particular needs of their followers (McClelland, 1975, 1985; Spangler and House, 1991). They are accommodating and sympathetic (Koestner and McClelland, 1992) and try not to hurt others’ feelings (Weinberger et al., 2010). As leaders high in *n*Aff consider followers individually and are sensitive toward their specific needs (Kanungo and Mendonca, 1996), we assume:

*Hypothesis 2:* A leader’s *n*Aff positively relates to perceived concern for her or his followers’ needs.

This wish to accommodate one’s followers’ needs may induce particularistic judgments that other team members perceive as unfair (McClelland, 1985). Yet, the value of considerate behaviors with regards to leadership outcomes has been supported by meta-analytic evidence (Judge et al., 2004). Moreover, a growing body of research has underscored the positive impact of *n*Aff on leadership success. Against the backdrop of the ambiguous findings, Boyatzis (1972) reasoned that *n*Aff may manifest itself in two different types: affiliative assurance and affiliative interest. Leaders characterized by the assurance type of *n*Aff long for the approval of other people and fear to be left alone (Boyatzis, 1973). They are anxious about being rejected by their followers or superiors, avoid conflicts, and do not give negative feedback on followers’ work performance in order to assure strong and secure relationships (Boyatzis, 1979). By contrast, leaders high in affiliative interest are concerned with their followers’ needs, welfare, and development. They support and empower them and create an atmosphere of openness and interpersonal trust. These leaders seek to work toward organizational objectives together with their subordinates and—as they are able to see a person’s performance independent of the relationship they have—provide them with feedback (Boyatzis, 1979). Whereas affiliative assurance interferes with necessary leadership tasks, Boyatzis (1979) highlighted that a pronounced affiliative interest benefits effective leadership.

In the 1960s, studies conducted by Lawrence and Lorsch (1967) and Litwin and Siebrecht (1967) found that *n*Aff distinguishes effective from less effective integrative leaders. The jobs of integrators center on getting people to work together and resolving team conflicts (Lawrence and Lorsch, 1967; McClelland, 1985). Apparently, these tasks are congruent with the affiliative leader’s need to establish and maintain close social networks. Research has also shown that *n*Aff positively relates to the work performance of first-line supervisors and their followers’ job satisfaction (Cornelius and Lane, 1984) as well as to a team’s collective performance (Kirkpatrick et al., 2002). These findings have variously been reduced to the specific context under investigation: the service industry. Companies in this area depend on establishing and maintaining good customer relations (Solomon, 1986); thus, *n*Aff proves to be highly valuable in this sector. However, affiliative concerns are relevant in all types of contemporary organizations (Spangler et al., 2014). Roughly 20 years ago, Burnham (1997) noticed that leadership demands were changing and so did the inner motivation of leaders. He highlighted a trend toward less hierarchical, more team-based structures that stress the equality of leaders and followers (Burnham, 1997). Such structures demand more democratic leadership actions (Spangler et al., 2014), lateral considerate management (Steinmann et al., 2015), and empathy from leaders (Burnham, 1997). Leaders nowadays act like coaches and care more about their followers’ personal and professional advancement (Steinmann et al., 2015). If leaders are high in *n*Aff, considerate or coaching leadership behaviors are motivationally energized and performed with greater persistence.

Given the ever-increasing diversification of teams (van Knippenberg and Schippers, 2007) stemming from globalized markets, increased occupational mobility, and aging workforces, leadership tasks have shifted toward integrative ones (see Nishii and Mayer, 2009), thus highlighting the concomitant rise of a highly pronounced *n*Aff into the status of an essential motivational asset. Furthermore, the growing importance of formal and informal networks inside and outside organizations in today’s competitive, globalized corporate environments also makes *n*Aff vital in effective leadership (Spangler et al., 2014). Leaders need to initiate, maintain, and restore interactions with peers and superiors. They have to cooperate with suppliers, customers, and other stakeholders and are expected to encourage collaboration and teamwork (Spangler et al., 2014). As a result, networking has become a critical determinant of career success (Ng et al., 2005). Being behaviors necessary in building, maintaining, and using relationships (Wolff and Moser, 2009), networking corresponds with the behavioral manifestation of *n*Aff. Finally, in current times of vocational uncertainty, followers need to stay committed to organizations and their goals (Lim and Ployhart, 2004). If leaders are aware of their followers’ needs and individually care for them, like leaders high in *n*Aff do, their followers have a higher degree of commitment (Rafferty and Griffin, 2006).

A study recently conducted by Steinmann et al. (2015) has confirmed these considerations. Specifically, it found that *n*Aff contributes to leaders’ career success and followers’ goal attainment across various types of organizations. However, an interacting socialized power motive was also needed for this effect to occur. This finding supports the assumption that affiliative concerns have developed into indispensable motivational ingredients and necessary add-ons (Spangler et al., 2014) of leadership success.

### Interactive Effects of Implicit Motives

Although research in this field has been interested in how motive combinations affect a person’s behavior since its beginning, only recently have these combinations been analyzed in terms of interactive effects (Steinmann et al., 2015). Previous studies tended to draw on methodologically debatable dichotomous configurations such as the LMP. The results of this recent analysis corroborate the notion that implicit motives indeed have an interactive effect and modulate each other. Based on this finding, we argue that *n*Ach moderates the effect of the leaders’ *n*Pow on indicators of effective leadership. Driving leaders to seek pleasure from influencing others, a distinct *n*Pow still is *the* essential motivational foundation for a leader’s success. However, current organizational contexts require leaders to also be innovative and competitive and to take important business decisions. Therefore, *n*Ach has become a necessary condition that makes power-motivated leaders more successful. While high *n*Pow drives leaders to motivate, coordinate, and influence followers (Spangler and House, 1991), *n*Ach channels these behaviors such that the leaders’ influence is oriented toward task completion, long-term involvement, excellence, and innovativeness—thus organizational success. Although leaders who are solely motivated by *n*Ach would generally avoid necessary leadership tasks, the underlying power motive drives them to seek pleasure from regulating others.

#### The Moderating Effect of *n*Aff on Leadership Success

Activity inhibition plays a critical role in leadership because it channels the leader’s display of power into beneficial pathways. However, research has failed to clearly show that the interaction between *n*Pow and AI contributes to leadership performance (Spangler and House, 1991; De Hoogh et al., 2005). Instead of referring to AI in elaborating on the successful use of power, both Winter (1973) and McClelland (1975) highlighted the need for affiliation to counterbalance the effects of excessive power motivation. Winter reasoned that leaders high in *n*Pow may end up leading in a dictatorial manner if they are not concurrently motivated by the need for affiliation. He thus assigned a central role to *n*Aff in leadership by arguing that it has the ability to channel the use of power into more effective pathways. Based on current leadership demands and increasing evidence on the value of *n*Aff in the leadership context, we take up this line of reasoning and ascribe a moderating function in predicting successful leadership to *n*Aff. Exceeding beyond the impact of AI on individual motives, we assume that a leader’s *n*Aff modulates the interaction of *n*Pow and *n*Ach. We posit that contingent on high *n*Ach, *n*Pow more closely relates to leadership success if concurrently moderated by high *n*Aff. In this interplay, the distinct need for affiliation drives leaders to be attentive to their followers’ goals and projects, to support their vocational and personal development, and to initiate, maintain, and restore relations that are beneficial to the advancement of their careers and the achievement of unique accomplishments. Therefore, an increase in followers’ satisfaction and performance as well as leaders’ career success is contingent on this motive.

##### Affecting followers’ satisfaction

Modulated by high *n*Ach, high *n*Pow motivates leaders to use their impact to attain excellent performance and extraordinary goals. Although this focus on task completion positively relates to follower satisfaction, the relation between considerate leadership and follower satisfaction is even stronger because these behaviors better satisfy the followers’ needs (Judge et al., 2004). Leaders high in *n*Aff are more sensitive to these needs (Boyatzis, 1979). As these leaders are benevolent and accommodating (Koestner and McClelland, 1992), but at the same time try to maintain their followers’ affection (McClelland, 1975), they consider these needs while leading, thus increasing their followers’ satisfaction. Given the moderating effect of *n*Ach, the followers’ needs are satisfied in accordance with task affordances instead of ignoring organizational necessities. Under the condition of being high in *n*Aff, leaders allow their followers to bring in new ideas, involve them in the decision-making process, and consider them partners in attaining corporate goals (Kanungo and Mendonca, 1996). This type of power sharing equally raises their followers’ satisfaction (e.g., Kalshoven et al., 2011). The goals that leaders high in *n*Pow strive for may be of personal relevance only. Moderated by *n*Ach, this striving is directed at task-completion, irrespective of the costs for others. If they are high in a concern for others, on the other hand, the leaders’ goals relate to the good of the collective (Kanungo and Mendonca, 1996). Followers, whose leaders strive to maximize personal gains at all costs, have lower job satisfaction and less positive attitudes toward their leaders (Schyns and Schilling, 2013). By contrast, those of leaders whose behaviors are directed at higher-order goals that transcend self-interests are more satisfied with their jobs and leaders (e.g., Judge and Piccolo, 2004). As it drives leaders to take interest in others (Boyatzis, 1979), high *n*Aff also makes leaders more aware of and attuned to their followers’ strivings. If followers are allowed to pursue their personal goals, their subjective well-being is higher. When it comes to work goals, progress relates to an employee’s job satisfaction (Klug and Maier, 2015). Leaders high in *n*Aff permit followers to pursue such goals, and even lend support (Kanungo and Mendonca, 1996) and encourage their development (Steinmann et al., 2015). Followers are not only more satisfied with their jobs; they are also more satisfied with their leaders because they give them the freedom to pursue their own goals. Against this background, we assume:

*Hypothesis 3:* Contingent on high *n*Ach, a leader’s *n*Pow more closely relates to her or his followers’ (a) job satisfaction and (b) satisfaction with the leader if moderated by high compared to low *n*Aff.

##### Affecting followers’ performance

The task-orientation that leaders interactively motivated by *n*Pow and *n*Ach show is obviously associated with a group’s performance. However, research has again found that considerate leadership behaviors also relate to it (Judge et al., 2004). Contingent on high *n*Aff, leaders are considerate of their followers and foster their development (Boyatzis, 1979). They lend task-oriented support, but—based on their recognition of followers as having the potential to add to organizational objectives—also show confidence in their subordinates’ abilities and lend them encouragement (Kanungo and Mendonca, 1996). Task-oriented support involves the provision of resources which are instrumental in completing a job and thus benefits the followers’ job performance directly. The leaders’ confidence and assurance, on the other hand, strengthen their followers’ self-efficacy (Eden, 1990). As higher levels of self-efficacy relate to higher work performance (Stajkovic and Luthans, 1998; Judge and Bono, 2001), an interacting *n*Aff also indirectly contributes to enhancing the followers’ performance. Under the condition of being high in *n*Aff, leaders empower their followers psychologically (Seibert et al., 2011). Feeling empowered not only positively influences the in-role but also the contextual performance of individuals and entire teams (Seibert et al., 2011). As empowered followers are more confident in their abilities, more convinced they may make meaningful contributions to corporate goals, and have more of a feeling of personal mastery, they are likely to perform beyond the call of duty (Spreitzer, 2008). Affected by high *n*Ach, leaders driven by high *n*Pow engage in behaviors which exceed the demands of their role. If further moderated by high *n*Aff, these behaviors are directed at the welfare of the organization and its members, which turns leaders into role models. In addition, the consideration and empowerment provided by leaders high in *n*Aff may encourage their followers to reciprocate their support and affection (see Gouldner, 1960). Followers return their leaders’ investment not only by enhancing their work efforts, but also satisfy this moral obligation by showing organizational citizenship behavior (OCB; Settoon et al., 1996). Thus, we assume:

*Hypothesis 4:* Contingent on high *n*Ach, *n*Pow more closely relates to the followers’ (a) in-role performance and (b) OCB if moderated by high compared to low *n*Aff.

##### Affecting leaders’ career success

Meta-analytic evidence (Ng et al., 2005) has shown the amount of people one knows within the organization and the extent of networking an employee engages in is significantly related to her or his salary level and number of promotions. Whereas leaders high in *n*Pow may seek pleasure from broad social networks because they provide opportunities to influence others and may be considered a source of building one’s reputation, networks satisfy one’s *n*Ach by constituting a way of furthering the attainment of goals and getting ahead of others (Wolff and Moser, 2009). Although leaders high in *n*Pow and *n*Ach use their relationships to complete prestigious tasks more effectively, high *n*Aff drives leaders to first initiate them. It also makes them more attentive to social cues, motivates the initiation of interactions (Weinberger et al., 2010), and fosters an effortless learning of social networks (McClelland, 1985).

As those high in affiliation are pleasant to be around and foster reciprocal communication (Weinberger et al., 2010), this motive helps to maintain social ties. It is only when leaders are sensitive to their networking partners’ needs and concerns, when they reciprocate their efforts, and do not solely exploit them for their own good that mutually gratifying and thus lasting networks evolve (see Gouldner, 1960). As *n*Aff channels interactions within networks toward more reciprocation, it assists the leader in advancing her or his career on the long run. Leaders with a strong concern for others further strive to mutually share their thoughts and feelings with those around them (Weinberger et al., 2010). Thus, they may be assumed to also be more aware of their own superiors’ strivings. Based on the interplay between high *n*Pow and *n*Ach, leaders may use this knowledge strategically to do their superiors favors they appreciate. Doing favors is an important political skill (Wayne et al., 1997) that is instrumental in advancing one’s career (Ng et al., 2005). As high *n*Aff contributes to both networking behavior and political skills, we assume:

*Hypothesis 5:* Contingent on high *n*Ach, *n*Pow more closely relates to leaders’ career success if moderated by high compared to low *n*Aff.

##### Affecting perceived leadership behavior

We assume that high *n*Pow moderated by high *n*Ach expresses itself in more transformational leadership behaviors if the leaders’ *n*Aff is equally high. Modulated by an interplaying *n*Ach, their concern for influencing others drives power-motivated leaders to expand their aspirations to followers and to align subordinates’ actions to excellence. They enforce high performance expectations, but by illustrating the goals they are striving for, they also convince their followers of the value of efforts. If high *n*Aff adds to this interplay, leaders develop a compelling vision that also benefits the group. Based on the affiliative leaders’ awareness of their followers’ inner lives (Weinberger et al., 2010), this vision takes up and elevates the followers’ needs and values. When they intertwine vision and self-concepts in such a way, affiliative leaders provide the work with meaning and inspire their followers (Bass and Avolio, 1994). Given their concern with avoiding routines and improving things (McClelland, 1985), the interplaying *n*Ach drives leaders high in *n*Pow to prompt their followers to also challenge assumptions, reframe problems, and approach situations in new ways. Thus, they stimulate them intellectually (Bass and Avolio, 1994). Moreover, contingent on high *n*Aff, leaders are confident in their followers and create an atmosphere of trust (Boyatzis, 1979). Followers need not fear criticism if they make mistakes while applying these new procedures. Transformational leaders are role models with whom followers identify (Bass and Avolio, 1994). The desire to emulate one’s leader is rooted in the leaders’ placing others’ needs above their own (Bass and Avolio, 1994). Because it keeps leaders sensitive to followers’ needs and arouses altruism (Kanungo and Mendonca, 1996), high *n*Aff not only contributes to such idealized influence, but also energizes individualized consideration. Leaders that are driven by *n*Aff are aware of their followers’ needs for self-actualization and create opportunities for personal growth (Boyatzis, 1979). Acting as coaches, these leaders delegate tasks, foster a supportive climate, show confidence in their followers, and use their power to develop followers to higher levels of potential (Bass and Avolio, 1994). As putting followers’ needs beyond one’s own self-interests essentially depends on the leaders’ awareness of these needs (see Avolio et al., 1991), we assume:

*Hypothesis 6:* Contingent on high *n*Ach, *n*Pow more closely relates to perceptions of transformational leadership if moderated by high compared to low *n*Aff.

## Materials and Methods

### Procedure

To examine these hypotheses, we developed two distinct surveys. The first was designed to assess the leaders’ implicit motives and career success. The second assessed the followers’ perceptions of leadership behavior as well as their performance and degree of satisfaction with their jobs and leaders. We conducted the study as an online survey to be able to address a wide range of potential participants. For the same reason, we contacted both leaders and followers in recruiting participants. We contacted employees in leading or non-leading positions we knew personally and invited them to take part in our study. However, we also approached potential participants in (virtual) business networks and promoted our study on appropriate platforms. To further spread the survey, these leaders and followers were asked to forward the link to colleagues, friends, and anyone else who would be interested in the topic.

The study was introduced as a research project on the motivation of leaders. The leaders were told that participation required writing stories that corresponded to pictured situations. The followers were informed that the study entailed questions on their leader’s leadership behavior as well as on their own work-related behavior and satisfaction. Depending on whether the leader or one of the followers completed the survey first, the participants were asked to forward the link to the survey to either a follower or their immediate leader. To mitigate the effect of liking, we asked the leaders to forward the link to the follower with whom they felt they worked the closest (see De Hoogh et al., 2005). To match data sets, the leaders and followers developed a pre-structured code unique to the dyad. Since we approached leaders and followers simultaneously, and as leaders may have forwarded the link to the follower survey to several employees, more than one follower of a given leader could have participated in the study. If more than one follower finished the survey, we drew on the data of that follower who first completed the questionnaire and excluded the remaining data sets.

### Ethics Statement

Before starting the data collection, we consulted our university’s ethics committee and answered its application questionnaire in order to evaluate whether the study complied with common ethical standards. As we did not employ any method that deviated from legal regulations or the ethical guidelines of the German Association of Psychology, no further steps were needed to ensure the ethical innocuousness of the study. As personal data was not assessed in our surveys, we did not obtain written informed consent of the participants in order to protect their anonymity. Yet, we emphasized that by closing their internet browser participants could abandon the survey at any time. Participants were assured that incomplete data would be deleted and would not enter the analyses. No vulnerable populations have been involved.

### Participants

In sum, 108 leaders and 94 followers finished the online survey. Though, we had to exclude some of the participants because four leaders had been evaluated by more than one follower (exclusion of seven followers), the leaders or followers could not be assigned a counterpart (exclusion of 25 leaders and four followers), or either the leader or the follower did not fully complete the questionnaire (exclusion of 13 leader-follower dyads). Thus, the analyses were based on *N* = 70 leader-follower dyads. Longitudinal studies relating leaders’ motives to their promotion used samples of more than 200 leaders (McClelland and Boyatzis, 1982; Jacobs and McClelland, 1994); however, those based on leader-follower dyads commonly drew on smaller samples that spanned between 28 CEOs and 56 of their followers in the study by Delbecq et al. (2013) to 82 leaders with 140 followers in Kirkpatrick et al.’s (2002) study. Our sample hence lies within the range of similar studies.

Of the final 70 leaders, 21.4% were female and 78.6% were male. Their age ranged between 31 and 63 years (*M* = 45.86; *SD* = 8.23). Nearly two thirds (65.2%) held university degrees and 21.7% had graduated from professional academies. The average level of work experience was about 24 years (*M* = 23.73; *SD* = 10.17). In terms of length in a leadership position, the leaders had averaged a bit over 13 years (*M* = 13.21; *SD* = 9.21) and had served an average of almost 10 years (*M* = 9.56; *SD* = 7.91) in their current position. On average, they led 12 followers (*M* = 12.07; *SD* = 11.49), ranging from one to 60. More than a third (34.8%) indicated that they were general managers or members of executive boards.

The final sample of followers was made up of 47.8% females and 52.2% males. Their age ranged between 21 and 59 years (*M* = 39.87; *SD* = 9.91). Among the followers, 41.4% had obtained a university or polytechnic degree, 22.9% had completed an apprenticeship, 17.1% had studied at vocational colleges, and 11.4% graduated from professional academies. Their work experience exceeded 18 years (*M* = 18.36; *SD* = 11.47). As for employment type, the followers worked either full-time or part-time. The majority had a weekly working time of 31–40 h (68.6%), 12.9% of more than 40 h, and 11.4% worked in-between 21 and 30 h. The remaining followers worked less than 20 h a week.

The participants worked in various industrial sectors with the manufacturing sector (53.6%), service sector (13%), and non-profit organizations (8.7%) being the ones most represented. Slightly more than half of the people were employed in small and medium-sized businesses with less than 250 employees (50.7%), about one quarter (26.1%) in large-scale enterprises with more than 1,000 employees. Their jobs were mainly located in the commercial (50.7%) or technical sector (15.9%) of the organization. On average, the leaders and followers had been working together for about 6 years (*M* = 5.87; *SD* = 5.31).

### Measures

#### Implicit Motives

We measured the leaders’ implicit motives using the Picture Story Exercise (PSE; see McClelland et al., 1989), a projective measure commonly used to capture a person’s inner strivings. As they are not subject to introspection, motive scores cannot be obtained through self-report questionnaires. However, they can be derived by coding the content of imaginative stories people write in response to ambiguous pictures (see Schultheiss and Pang, 2007). As we aimed to assess *n*Pow, *n*Ach, and *n*Aff, we used the pictures couple by a river, nightclub scene, women in laboratory, ship captain, trapeze artists, and boxer as recommended by Pang and Schultheiss (2005). The pictures were presented randomly for 10 s each. According to the instruction by Smith et al. (1992), the participants were then invited to write a story about the people illustrated (e.g., about their thoughts and feelings, what happened before, and what would happen next). In order to derive motive scores, the participants need to produce sufficient written material in response to the pictures. Smith et al. (1992) recommend that, to obtain a sound coding, a participant has to elaborate on at least two thirds of the pictures with 30 words per story being the minimum word count. The leaders were asked to spend 4 min to write each imaginative story. To encourage them to write, it was only possible to continue to the next page of the survey after 90 s had elapsed. No maximum time limit was imposed.

Two well-trained scorers (exceeding category agreement of 85%) scored the stories of the 108 leaders who finished the survey according to Winter’s (1994) coding manual for implicit motives. *n*Pow was scored if the leaders referred to strong and energetic actions that influenced others or the world at large. *n*Ach was scored if the leaders’ stories positively evaluated performance, mentioned successful competition, or hinted at unique accomplishments. Finally, *n*Aff was scored if the stories indicated positive feelings toward others, centered on companionate activities, but also if they alluded to feelings of sadness due to being separated from others (Winter, 1994). Activity inhibition was measured by counting how frequently leaders used the word “not” in their stories. One quarter of the stories (stories of 27 leaders) was initially rated by both scorers. Interrater reliability was *r* = 0.96 for *n*Pow, *r* = 0.97 for *n*Ach, and *r* = 0.98 for *n*Aff (*p* < 0.001). Discrepancies were discussed and further coding guidelines developed (see Schultheiss and Pang, 2007). The stories of the remaining 81 leaders were then distributed between the two scorers. Among these leaders, nine did not write enough for a sound coding of their implicit motives and were subsequently excluded.

The leaders entering the analyses wrote 387 words on average (*M* = 387.03; *SD* = 191.00), with 134 being the minimum and 1,285 the maximum values. Motive imagery ranged from 0 to 13 (*M* = 3.70; *SD* = 2.49) for *n*Pow, from 0 to 10 (*M* = 2.70; *SD* = 1.98) for *n*Ach, and from 0 to 12 (*M* = 4.16; *SD* = 2.50) for *n*Aff. AI ranged between 0 and 14 (*M* = 2.62; *SD* = 2.91). As the total number of motive imagery and AI significantly related to the word count (*r* = 0.69 for *n*Pow, *r* = 0.62 for *n*Ach, *r* = 0.68 for *n*Aff, and *r* = 0.64 for AI, all *p*s < 0.001) we adjusted the motive scores for protocol length using regression analyses (Schultheiss and Pang, 2007). Residualized and *z*-standardized motive scores were entered in all subsequent analyses.

#### Leadership Behavior

The followers’ perceptions of passive and transformational leadership were assessed using the German version (Felfe and Goihl, 2002) of the Multifactor Leadership Questionnaire (MLQ; Bass and Avolio, 1995). Passive leadership was measured with eight items (e.g., “My direct supervisor waits for things to go wrong before taking action”), transformational leadership with 20 items (e.g., “My direct supervisor talks enthusiastically about what needs to be accomplished”). On a five-point response scale ranging from 1 = *never* to 5 = *almost always*, the followers stated how often their leaders showed the behaviors illustrated. Passive leadership was assessed with an internal consistency of α = 0.84; transformational leadership with an internal consistency of α = 0.93. To determine perceptions of the leaders’ concern for their followers’ needs, we used three items developed by Rafferty and Griffin (2006; e.g., “My direct supervisor considers my personal feelings when implementing actions that will affect me”). Based on a five-point response scale ranging from 1 = *not at all* to 5 = *entirely*, the followers indicated the extent to which the statements applied to their leaders. Reliability of the scale was α = 0.87.

#### Satisfaction

Job satisfaction was measured with a short version of Neuberger and Allerbeck’s (1978) Job Description Form. On a seven-point Kunin-scale, the followers quantified how satisfied they were with regard to various facets of their jobs (e.g., colleagues, promotion opportunities, and work conditions). Reliability of the seven-item scale was α = 0.79. Their satisfaction with their leader was assessed using the same-named scale of the MLQ (Bass and Avolio, 1995; Felfe and Goihl, 2002). Its two items (e.g., “My direct supervisor uses methods of leadership that are satisfying”) were evaluated on a five-point response scale ranging from 1 = *never* to 5 = *almost always*. Taken together, these items indicate how frequently the followers were satisfied with their leaders. The scale had an internal consistency of α = 0.90.

#### In-Role Performance and OCB

In-role performance and OCB were measured using an instrument developed by Staufenbiel and Hartz (2000). Based on a seven-point response scale ranging from 1 = *not at all* to 7 = *entirely*, the followers indicated the extent to which the items pertained to themselves. Assessing in-role performance, five items are commonly evaluated (e.g., “I meet the obligations defined in the job specification”). As Cronbach’s α was dissatisfying for the five-item scale in the present study (α = 0.68), we deleted one of the items (i.e., “I neglect things that are part of my duties”) to increase reliability to α = 0.74. OCB was measured with 20 items (e.g., “I help others if they have heavy workloads”) and had a reliability of α = 0.80.

#### Career Success

Because it is the most prominent indicator of objective career success (Ng et al., 2005), we assessed developments in a leader’s income. In a single item, leaders rated how their income had developed during the previous 12 months (see Steinmann et al., 2015). The five-point response scale ranged from 1 = *substantial decrease* to 5 = *intense increase*.

## Results

### Preliminary Analyses

Before testing our assumptions, we conducted several analyses to ensure those participants within the final sample and those who were excluded from the analyses did not exhibit any systematic difference in terms of demographics or the relevant constructs. Accounting for multiple comparisons, we Bonferroni-adjusted the alpha level for the number of tests needed.

First, we examined whether the leaders entering the final sample significantly differed from those who had been dropped because they lacked a follower or were part of a dyad in which one of the surveys had not been fully completed. The analyses did not reveal any significant difference between the groups concerning their age, gender, work or leadership experience, tenure in the current leadership position, hierarchical level, number of followers, educational background, functional area, industrial sector, size of the organization, or developments in income (all *p*s > 0.004). With regard to followers, we also analyzed whether any systematic variation between those in the final sample and those who were excluded might distort the results of the main analyses. The analyses confirmed that both groups of followers did not differ in terms of age, gender, work experience, educational background, or weekly working time (all *p*s > 0.01).

Next, we considered the followers’ evaluations of their leaders’ behaviors, their own work performance, and their work-related satisfaction. To ensure no significant connection between the followers’ assessment and the leaders’ study participation existed, we compared the data of the followers whose leaders took part in the survey with those of followers whose leaders did not. Ratings of transformational and passive leadership, the leaders’ concern for their followers’ needs, as well as followers’ performance, OCB, and both satisfaction indicators did not significantly vary (all *p*s > 0.007) between either group of leaders.

Finally, we concentrated on leaders who produced sufficient written material for a sound coding of their implicit motives. In this group we analyzed whether differences in motive imagery and activity inhibition emerged between those who were evaluated and those whose followers did not participate in the study. Across the groups, we did not find any systematic difference in the leaders’ *n*Pow, *n*Ach, *n*Aff, or AI (all *p*s > 0.013).

### Examination of Hypotheses

#### The Behavioral Manifestation of *n*Ach and *n*Aff

Next, we went to validate our hypotheses. Hypothesis 1 assumed that leaders’ *n*Ach is positively associated with their followers’ perceptions of passive leadership. Hypothesis 2 postulated a significant positive relation between *n*Aff and perceptions of the leaders’ concern for their followers’ needs. To test these assumptions, we related word count adjusted and *z*-standardized motive scores to the followers’ assessment of their leaders’ leadership behavior. **Table 1** displays the intercorrelations of all variables in the study. It illustrates that the leaders’ *n*Ach only tended to relate to their followers’ perceptions of passive leadership behavior (*r* = 0.21, *p* < 0.10). The leaders’ *n*Aff was positively and significantly associated with the followers’ evaluation of their leaders’ concern (*r* = 0.25, *p* < 0.05), which fully supports our assumption.

**Table 1:** Means, standard deviations, and intercorrelations among the variables in the study.

	*M*	*SD*	(1)	(2)	(3)	(4)	(5)	(6)	(7)	(8)	(9)	(10)	(11)
(1) *n*Pow	0.00	1.00											
(2) *n*Ach	0.00	1.00	0.04										
(3) *n*Aff	0.00	1.00	-0.07	-0.02									
(4) AI	0.00	1.00	-0.03	-0.14	0.21^†^								
(5) Passive leadership	1.99	0.66	0.03	0.21^†^	-0.04	0.02							
(6) Concern for followers’ needs	3.93	0.80	0.11	-0.19	0.25^*^	-0.06	-0.64^***^						
(7) Job satisfaction	5.57	0.83	0.01	-0.02	0.14	-0.32^**^	-0.55^***^	0.62^***^					
(8) Satisfaction with the leader	4.34	0.76	0.09	-0.20^†^	0.06	-0.18	-0.66^***^	0.64^***^	0.71^***^				
(9) In-role performance	6.10	0.69	-0.03	0.02	0.12	0.04	-0.30^*^	0.03	0.21^†^	0.15			
(10) OCB	5.61	0.52	-0.18	-0.12	0.15	0.16	-0.27^*^	0.17	0.31^**^	0.38^**^	0.27^*^		
(11) Career success	3.53	0.72	-0.11	-0.01	-0.18	0.11	0.06	-0.10	-0.04	-0.07	-0.03	-0.12	
(12) Transformational leadership	3.96	0.61	0.09	-0.05	0.14	-0.15	-0.70^***^	0.77^***^	0.67^***^	0.71^***^	0.02	0.22^†^	-0.03

*^∗∗∗^*p* < 0.001, ^∗∗^*p* < 0.01, ^∗^*p* < 0.05, ^†^*p* < 0.10.*

Although we did not find any significant association with regard to *n*Pow, there tended to be a positive relation among *n*Aff and AI (*r* = 0.21, *p* < 0.10) and a negative among *n*Ach and followers’ satisfaction with the leader (*r* = -0.20; *p* < 0.10). Furthermore, **Table 1** shows that the leaders’ AI significantly and negatively related to their followers’ job satisfaction (*r* = -0.32, *p* < 0.01).

#### The Moderating Effect of *n*Aff on Leadership Success and Leadership Behavior

To examine the moderating effect of the need for affiliation on the interplay of power and achievement, we computed all two-way interaction terms and the three-way interaction term among leaders’ *n*Pow, *n*Ach, and *n*Aff based on the residualized and *z*-standardized motive scores. As we were interested in testing the moderating effect of *n*Aff beyond the impact of AI on motives, we included AI and the related two-way interactions into our hierarchical regression analyses. For all outcomes, the motives and AI were first entered into the regression, followed by the two-way interactions among *n*Pow, *n*Ach, *n*Aff, and AI, and the three-way interaction term of *n*Pow, *n*Ach, and *n*Aff in the last step of the regression.

**FIGURE 1:**
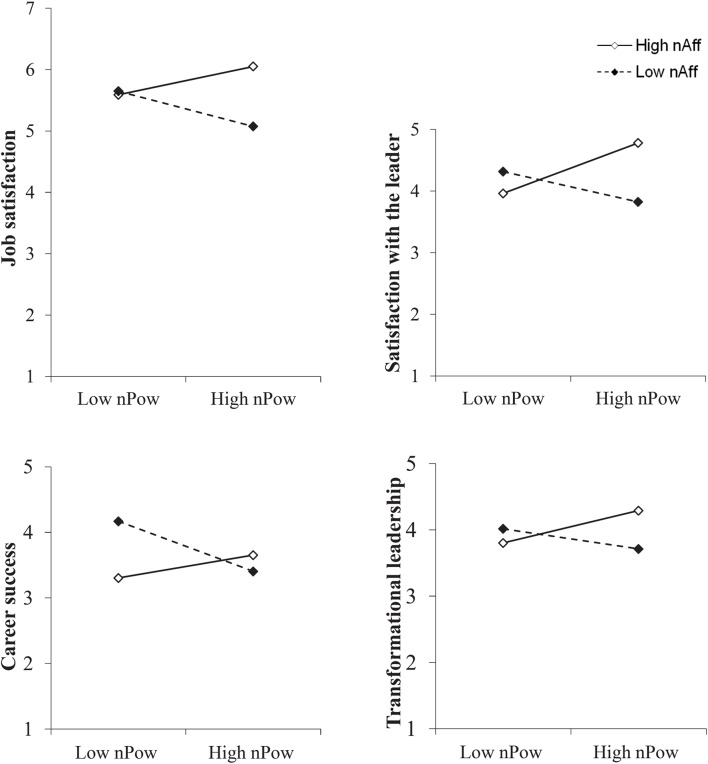
**Regression slopes for the relationship between *n*Pow and the followers’ job satisfaction, their satisfaction with their leader, the leaders’ career success and perceptions of transformational leadership at high and low levels of *n*Aff with *n*Ach held constant at high levels**.

Hypothesis 3 postulated that contingent on high *n*Ach, the association between a leader’s *n*Pow and her or his followers’ (a) job satisfaction as well as (b) satisfaction with the leader would be closer if moderated by high compared to low *n*Aff. The analyses showed that the three-way interaction between *n*Pow, *n*Ach, and *n*Aff did in fact significantly account for additional variance in both satisfaction indicators (**Table 2**). With regard to job satisfaction, the three-way interaction was significant on the 1%-level and additionally explained 10.2% of the variance. As for the followers’ satisfaction with their leader, it contributed to the explanation of another 9.7% of the variance (*p* < 0.01). To further analyze the specific form of the three-way interaction, we plotted the slopes of interest (**Figure 1**). In line with our assumptions, *n*Pow is displayed on the *x*-axis and the slopes are plotted for high levels and low levels of *n*Aff, with *n*Ach held constant at high levels. The plot indicated the relation to be closer if high *n*Aff added to the interplay. To further give evidence on the promoting effect of high *n*Aff, we applied Dawson and Richter’s (2006) slope difference test and statistically examined whether a difference between the slopes emerged. For each outcome, the slope difference tests showed a significantly closer relation if moderated by high *n*Aff (**Table 3**).

**Table 2:** Hierarchical regression analyses predicting followers’ satisfaction and work performance, leaders’ career success, and perceptions of transformational leadership behavior from implicit motives and activity inhibition.

	Job satisfaction				Satisfaction with the leader				In-role performance			
			
Variable	*B*	*SE B*	β	*t*	*B*	*SE B*	β	*t*	*B*	*SE B*	β	*t*
Step 1												
*n*Pow	0.04	0.09	0.05	0.48	0.12	0.08	0.15	1.42	0.02	0.09	0.03	0.23
*n*Ach	-0.03	0.09	-0.03	-0.30	-0.16	0.08	-0.21	-1.88†	0.00	0.09	0.00	0.03
*n*Aff	0.16	0.10	0.20	1.60	0.08	0.09	0.11	0.91	0.14	0.10	0.21	1.44
AI	-0.22	0.10	-0.27	-2.31^∗^	-0.12	0.09	-0.15	-1.35	0.04	0.09	0.06	0.43
Step 2												
*n*Pow × *n*Ach	-0.07	0.09	-0.10	-0.84	-0.04	0.08	-0.05	-0.46	-0.04	0.08	-0.07	-0.49
*n*Pow × *n*Aff	0.04	0.11	0.04	0.33	0.13	0.09	0.15	1.34	0.13	0.10	0.18	1.32
*n*Ach × *n*Aff	0.07	0.10	0.09	0.65	0.07	0.09	0.10	0.77	0.07	0.10	0.11	0.69
*n*Pow × AI	0.17	0.09	0.24	1.95†	0.28	0.08	0.42	3.59^∗∗^	-0.02	0.08	-0.03	-0.21
*n*Ach × AI	0.02	0.09	0.02	0.18	0.02	0.08	0.03	0.26	-0.03	0.09	-0.04	-0.29
*n*Aff × AI	-0.07	0.11	-0.08	-0.64	0.01	0.10	0.02	0.13	-0.11	0.11	-0.15	-1.02
Step 3												
*n*Pow × *n*Ach × *n*Aff	0.22	0.08	0.37	2.95^∗∗^	0.20	0.07	0.36	3.00^∗∗^	-0.02	0.07	-0.03	-0.24
R^2^				0.32^∗^				0.37^∗∗^				0.11
ΔR^2^				0.10^∗∗^				0.10^∗∗^				0.01

	OCB				Career success^a^				Transformational leadership			
			
Variable	*B*	*SE B*	β	*t*	*B*	*SE B*	β	*t*	*B*	*SE B*	β	*t*

Step 1												
*n*Pow	-0.07	0.07	-0.13	-1.05	-0.07	0.09	-0.09	-0.77	0.08	0.07	0.14	1.16
*n*Ach	-0.03	0.07	-0.05	-0.37	0.05	0.09	0.07	0.56	-0.04	0.07	-0.06	-0.50
*n*Aff	0.06	0.07	0.12	0.85	-0.16	0.09	-0.22	-1.70^†^	0.09	0.08	0.15	1.20
AI	0.08	0.07	0.16	1.20	0.19	0.09	0.26	2.12^∗^	-0.07	0.07	-0.11	-0.94
Step 2												
*n*Pow × *n*Ach	-0.05	0.06	-0.11	-0.81	-0.04	0.08	-0.07	-0.51	-0.04	0.07	-0.07	-0.56
*n*Pow × *n*Aff	0.10	0.08	0.18	1.39	0.13	0.10	0.17	1.38	0.06	0.08	0.09	0.79
*n*Ach × *n*Aff	0.01	0.07	0.02	0.16	0.00	0.09	0.01	0.03	0.00	0.08	-0.01	-0.04
*n*Pow × AI	-0.01	0.06	-0.03	-0.23	-0.14	0.08	-0.23	-1.80^†^	0.21	0.07	0.39	3.13^∗∗^
*n*Ach × AI	0.04	0.06	0.08	0.62	-0.06	0.08	-0.10	-0.80	0.04	0.07	0.07	0.59
*n*Aff × AI	-0.02	0.08	-0.03	-0.20	-0.23	0.10	-0.30	-2.21^∗^	-0.03	0.09	-0.04	-0.31
Step 3												
*n*Pow × *n*Ach × *n*Aff	0.06	0.05	0.15	1.06	0.15	0.07	0.28	2.16^∗^	0.14	0.06	0.30	2.34^∗^
R^2^				0.15				0.29^∗^				0.27^∗^
ΔR^2^				0.02				0.06^∗^				0.07^∗^


*Regression weights and ΔR^2^ are taken from the last step of the hierarchical regression analyses. ^a^The analysis predicting career success is based on a sample of N = 68.*

*^∗∗∗^*p* < 0.001, ^∗∗^*p* < 0.01, ^∗^*p* < 0.05, ^†^*p* < 0.10.*

**Table 3:** Results of the slope difference tests comparing high and low levels of *n*Aff within the three-way interaction of *n*Pow, *n*Ach, and *n*Aff.

Outcome variable	*t*
Job satisfaction	2.12^∗^
Satisfaction with the leader	2.76^∗∗^
Career success^a^	2.35^∗^
Transformational leadership	2.09^∗^


*^a^The slope difference test relied on a sample of *N* = 68 for this outcome. ^∗∗^*p* < 0.01, ^∗^*p* < 0.05.*

Hypothesis 4 postulated that contingent on high *n*Ach, the relation between a leader’s *n*Pow and her or his followers’ (a) in-role performance and (b) OCB would be closer if modulated by high *n*Aff. Hypothesis 5 assumed the same moderating effect with regard to the leaders’ career success. Concerning the followers’ performance, the results of hierarchical regression analyses did not support our assumptions (**Table 2**). Neither in in-role performance nor in OCB did the three-way interaction term significantly account for variance increments (*p* > 0.10). With respect to career success, the interplay of *n*Pow, *n*Ach, and *n*Aff was significant (**Table 2**; *p* < 0.05). A visible inspection of the simple slopes for high and low levels of *n*Aff (**Figure 1**) suggested that the association was closer under the condition of high *n*Aff. Slope difference tests statistically supported the visual indication (**Table 3**). Due to missing data, the analyses of career success relied on a sample of *N* = 68 leader-follower dyads.

Finally, we assumed the interplay of high *n*Pow, *n*Ach, and *n*Aff would also be associated with a certain leadership style. Hypothesis 6 stated that under the condition of high *n*Ach, *n*Pow and perceived transformational leadership would be more closely related if high *n*Aff added to this interplay. A hierarchical regression analysis supported this assumption. On a 5%-level, the three-way interaction term accounted for additional variance. The degree of incrementally explained variance amounted to 6.9% (**Table 2**). Slope difference tests showed that given high levels of *n*Ach, the relation between *n*Pow and perceived transformational leadership was significantly closer if a leader’s *n*Aff was high (**Table 3**).

### Exploratory Analyses

We assume that to influence their followers and stimulate their performance, or affect their attitudes, a leader’s implicit motives need to manifest themselves in observable actions (see De Hoogh et al., 2005). As the interplay between *n*Pow, *n*Ach, and *n*Aff relates to perceptions of transformational leadership, we explored whether these behaviors transmit the effect on outcomes. Given that the three-way interaction did not account for variance increments in the followers’ performance and that transformational leadership did not relate to career success (**Table 1**), we limited the analyses to satisfaction indicators. Associations between transformational leadership and the followers’ job satisfaction (*r* = 0.67) as well as their satisfaction with their leader (*r* = 0.71, both *p* < 0.001) were significant. Supporting a mediator function of transformational leadership, the effect of the three-way interaction on job satisfaction (*B* = 0.12, *p* = 0.07) or satisfaction with the leader (*B* = 0.11, *p* = 0.07) diminished when, in addition to the interplay between *n*Pow, *n*Ach, and *n*Aff, transformational leadership was entered in the last step of hierarchical regression analyses. To refine the analyses, we tested mediation by using Hayes (2013) PROCESS-macro for SPSS. PROCESS allows for the examination of conditional indirect effects, estimates the indirect effect of higher-order interactions, and bootstraps its confidence intervals. Deviating from the conclusion based on Baron and Kenny’s (1986) procedure, the analyses in PROCESS corroborated that neither on job satisfaction (*b* = 0.104, CI [-0.007, 0.264]) nor on followers’ satisfaction with their leader (*b* = 0.097, CI [-0.015, 0.242]) was the effect of the three-way interaction transmitted by transformational leadership.

## Discussion

Integrating research on the contribution of *n*Aff to successful leadership with evidence on the interplay between implicit motives, the present study intended to demonstrate that under the condition of high *n*Ach, *n*Pow more closely relates to leadership success if a distinct *n*Aff adds to this interplay. Moreover, we sought to relate implicit motives and their interaction to leadership behaviors. We aimed to further illuminate the interaction between motives, to promote the value of affiliative interests in leading, and to advance knowledge on the behavioral manifestation of implicit motives in leadership.

### The Behavioral Manifestation of *n*Ach and *n*Aff

Based on the characteristics ascribed to people high in *n*Ach and in *n*Aff as well as on the behaviors they seek pleasure from, we assumed that the leaders’ *n*Ach manifests itself in the form of passive leadership and that their *n*Aff would appear as a concern for their followers’ needs. Considering *n*Aff by itself, the followers indicated that the leaders were more sensitive to their needs and more considerate of their feelings when taking actions. Thus, the leaders’ concern for others is not only an inner striving, but becomes visible in the way leaders interact with followers and does not go unnoticed by their subordinates. *n*Ach only marginally related to perceptions of passive leadership. Leaders who are solely driven by *n*Ach neglect essential leadership tasks (Spangler et al., 2014). Yet, they communicate performance evaluation standards, show disapproval if followers fail to meet them, and acknowledge improvements in the quality of work (Cornelius and Lane, 1984; Delbecq et al., 2013). Because they articulate expectations and recognize achievements, they actually take more leadership actions than passive leaders, who do not act until problems become severe or errors occur (Bass and Avolio, 1994). When followers make mistakes and do not complete tasks, they jeopardize the extraordinary achievements such leaders strive for; thus, leaders high in *n*Ach probably intervene more timely to prevent incidents and rather show active instead of passive management by exception (see Bass and Avolio, 1994).

### The Interactive Effect of *n*Pow, *n*Ach, and *n*Aff on Leadership Success

Based on high *n*Ach, we assumed that *n*Pow would more closely relate to indicators of leadership success if high *n*Aff adds to this interplay. The results of our analyses confirm that affiliation does indeed boost the relation to the followers’ satisfaction and the leaders’ career success. Followers are more satisfied contingent on high *n*Aff because this motive channels the leader’s strivings into socially acceptable ways and energizes follower-centered, supportive leadership behaviors. In an earlier study, Kolb and Boyatzis (1970) found a certain combination of the needs for power, achievement, and affiliation to result in effective helping behavior. Helping behavior relates to one’s intent to promote the development or improved functioning of others (Rogers, 1961). As leaders aim to develop their followers’ skills to improve their performance and foster their personal growth (Boyatzis, 1979), leader-follower-interactions have been considered a kind of helping relationship (Kolb and Boyatzis, 1979). Kolb and Boyatzis (1970) found people to be particularly effective helpers if they were moderately high in *n*Pow, *n*Ach, and *n*Aff. However, the study drew on MBA students who helped others to achieve personal change goals rather than on leaders. Although the authors state that helping behavior is best viewed in terms of an interactive effect of the three motives (Kolb and Boyatzis, 1979), analyses relied on main effects instead of considering their interplay as we did here. Looking at the three-way interaction, we further found leaders to be more successful in building their career if their *n*Aff was high compared to low. Given their interpersonal competence (Weinberger et al., 2010), affiliative leaders easily engage in public and social relations activities (Yukl, 2002) that can advance their careers (Ng et al., 2005). However, the plotting of the slopes reveals high *n*Ach modulated by low *n*Pow and low *n*Aff to be more important for the leaders’ career success. This finding is congruent with evidence found by McClelland and Franz (1992). Their study, which looked at a period of 10 years, revealed that even though work accomplishments depended on a socialized power motive, earned income was predicted by *n*Ach. Our results further suggest that Winter (1973) and McClelland (1975) were right in assuming that an excessive *n*Pow would threaten a leader’s success if her or his *n*Aff does not channel its negative impact. When we kept *n*Ach constant, increases in a leader’s *n*Pow fostered their followers’ satisfaction and perceptions of transformational leadership just as the leaders’ career success if high *n*Aff added to the interplay. When *n*Aff was low, relations did not only remain unchanged; increases in *n*Pow even diminished these favorable outcomes. Thus, *n*Aff does indeed have the ability to counteract the negative impacts of an excessive power motive and to channel the interplay of *n*Pow and *n*Ach into more successful pathways.

We did not find high *n*Aff boosted the association between *n*Pow and the followers’ in-role performance or OCB. We assumed that *n*Aff would enhance the followers’ performance by eliciting behaviors that raise their self-efficacy, empower them, and increase their moral obligation to repay the leaders’ concern. Studies have found empowering leadership to positively relate to followers’ performance (e.g., Srivastava et al., 2006; Vecchio et al., 2010). Yet, Cheong et al. (2016) recently showed that empowering leadership could also be a burden diminishing their performance. Relating to Langfred and Moye (2004), the authors reason that if followers are granted autonomy and job decision latitude, they would be cognitively distracted from performing tasks. This interference and the necessity to take inconvenient decisions cause stress in followers and negatively impact their performance (Cheong et al., 2016). An interacting *n*Aff makes leaders more aware of and confident in their followers’ abilities (Kanungo and Mendonca, 1996) and drives their desire to help others develop. It energizes the delegation of authority and the sharing of power. That way, high *n*Aff may increase followers’ work-related strain and ultimately undermine the positive impact we assumed. Cheong et al. (2016), however, found that, overall, empowering behaviors more strongly enabled than burdened followers. Rather than inducing stress, high *n*Aff may attenuate a leader’s drive to enforce performance expectations and monitor the goal pursuit. In addition, the empathy these leaders show and the concessions they make (Weinberger et al., 2010) may create the impression of being more lenient if followers do not make an effort. Thus, high *n*Aff may decrease followers’ performance if it adds to the interplay between *n*Pow and *n*Ach. In reciprocating their good will, the followers try to find ways to make their leaders notice their returns (see Gouldner, 1960). Leaders that are high in *n*Aff are concerned with developing their followers beyond job descriptions and they create a companionate atmosphere in teams. Therefore, contextual rather than in-role performance may be perceived as a better way of reciprocating. However, high *n*Aff did not enhance the followers’ OCB. The scale used in this study primarily assessed followers’ general compliance and proactivity toward the organization (Staufenbiel and Hartz, 2000). Followers, however, more likely reciprocate in interpersonal realms (Ilies et al., 2007). Given the concern with others that arises from high *n*Aff and the immediate positive interpersonal consequences such behavior entails, it is reasonable to assume a higher impact on individual-targeted OCB.

### The Behavioral Manifestation of the Interplay of *n*Pow, *n*Ach, and *n*Aff

As expected, the followers perceived their leaders’ behaviors to be more transformational contingent on high *n*Aff. The interplay of high *n*Pow, *n*Ach, and *n*Aff drives leaders to inspire their followers by challenging them and giving their actions meaning, to help them improve their abilities by intellectually stimulating them, to influence them ideally strengthening their desire to emulate the leader, and to consider them individually by lending support and acting as coach (Bass and Riggio, 2006). Thus, this interplay may be considered a motivational antecedent of transformational leadership. Studies have already found that *n*Pow relates to perceived or behavioral charisma in companies or politics (House et al., 1991; De Hoogh et al., 2005). Besides the fact that Delbecq et al. (2013) found a marginal negative relation between *n*Pow and charismatic leadership, charisma is only one component of transformational leadership (Bass and Riggio, 2006) and further behaviors add to eliciting performance beyond expectations (Bass, 1985). Whereas *n*Ach moderates the use of power toward intellectual stimulation and high performance expectations, *n*Aff channels the leaders’ behaviors into socially responsible ways. Going beyond the impact of AI, *n*Aff not only aligns leaders’ strivings and influence with corporate objectives, but energizes considerate behaviors directed at individual needs and personal growth. Such empathetic leadership is particularly important in involving followers’ self-concepts (Shamir et al., 1993).

Based on this finding, we explored whether these leadership behaviors transmit the effect of motives on followers’ satisfaction. Our results did not support a mediator function. Until now, transformational leadership has been shown to transmit the effect of leaders’ (emotional) intelligence (Hur et al., 2011; Cavazotte et al., 2012), positive psychological traits (Peterson et al., 2008), or Big Five personality traits (Lim and Ployhart, 2004; Cavazotte et al., 2012) onto the leaders’ effectiveness, the performance of groups or entire firms, or the team climate. The interplay between *n*Pow, *n*Ach, and *n*Aff exerts its influence on followers’ satisfaction in a different manner. Affiliative leaders are interpersonally warm and empathetic. As they are pleasant to be around (Weinberger et al., 2010), followers gravitate toward them. Their liking for the leader leads to more positive, higher quality leader-follower relationships, which, in turn, increase their satisfaction (Dulebohn et al., 2012). In this way, leaders’ motives may exert an influence on their followers’ attitudes by yielding certain characteristics rather than energizing particular leadership behaviors. With respect to career success, assuming mediation via leadership behavior would even imply a detour. Rather than depending on guiding followers, career advancement depends on the leaders’ abilities in initiating and using relationships that have the potential to maximize one’s own advantages (Wolff and Moser, 2009). Being proximal mediators of the effect of motives, networking behaviors are directly energized by the interplay between *n*Pow, *n*Ach, and *n*Aff. Thus, the outcomes, the behaviors or characteristics needed to achieve them, as well as the parties involved, have to be considered closely when regarding the manifestation of implicit motives in transmitting their effect on outcomes.

### Theoretical and Practical Implications

Research has been based on the assumption that several implicit motives energize a person’s behavior (McClelland, 1992). In studies on such combined effects, motives have commonly been condensed into typological configurations. Building off of earlier work (Spangler and House, 1991; Steinmann et al., 2015), this study applied a dimensional approach to motive combinations. Our results confirm that the needs for power, achievement, and affiliation interactively affect a person’s behavior and substantiate earlier findings on the interaction between implicit motives (Steinmann et al., 2015). Based on research, it has been reasoned that configurations yield personality portraits that differ from those evolving from the sum of single motives (McClelland, 1992). The present study shows that also leadership behaviors vary depending on the motives considered just as much as they are dependent on the interplay of these particular motives. Up to now, a modulating impact on the expression of motives has solely been attributed to activity inhibition and explicit personality constructs. Spangler et al. (2014) highlighted AI to also moderate the relation between *n*Ach or *n*Aff and leadership success. Research, however, examined the modulation of *n*Pow only. At times it has been reasoned that, rather than AI, a concern for responsibility would channel the use of power into socially acceptable ways (e.g., Winter, 1991). As high *n*Aff arouses a concern for others and makes leaders more sensitive to them, an interacting *n*Aff may foster the responsibility needed for effective leadership. Responsibility, though, involves morality, legality, and obligation (Winter, 1991), which do not necessarily accompany high *n*Aff. Exceeding the sticking to corporate procedures and the alignment to institutional goals that arise from AI (McClelland and Boyatzis, 1982), *n*Aff rather channels the expression of the interplay between *n*Pow and *n*Ach such that leaders are more confident in their followers’ abilities, individually care for them, promote their personal and vocational development, and create an atmosphere of working toward common goals, but at the same time also energizes behaviors conducive in establishing and maintaining strategic relationships used to maximize one’s advantages. Such behaviors may not be expected given the modulating effect of AI. Therefore, we postulate that while high AI restrains the unfiltered expression of motivational impulses inherent in a person thereby retaining the direction initially energized by that motive, interacting implicit motives rather add a new quality to the manifestation of other motives. Implicit motives and AI modulate the expression of motives so that different behaviors or characteristics evolve.

Besides these theoretical implications, the study entails initial implications for organizations. While in light of early research leaders would have been selected and promoted based on a social display of power, our results strengthen the importance of the need for affiliation in satisfying followers and advancing one’s career. As a concern with unique accomplishments and excellent performance has likewise developed into a driver of leadership success, practitioners charged with the selection of leaders have to reconsider and adapt HR instruments to do justice to the growing importance of *n*Ach and *n*Aff. When evaluating applicants’ conduct in assessment centers, for example, it is commonly assumed that people are less likely to make good leaders if they (e.g., in a leaderless group discussion) try to maintain harmonious relationships. In line with McClelland and Boyatzis (1982) and House et al. (1991), some might reason that such leaders would practice favoritism or refrain from enforcing necessary decisions. However, as current work environments require working with diverse teams in times of vocational uncertainty, considerate leader behaviors are vital in modern leadership (Lim and Ployhart, 2004; Homan and Greer, 2013). Evidence-based leadership competency models that incorporate behaviors energized by all three implicit motives (e.g., the LEaD model; Schmidt-Huber et al., 2014) may guide HR specialists in adjusting behavioral dimensions. Indicators of the behavioral manifestation of implicit motives and their interplay also have to be included in performance appraisal or 360-degree feedback to reveal motive dispositions. Although items on the manifestation of independent motives are quite easy to derive and align to the context of leadership, the way their interaction is reflected in a leader’s conduct and attributes deserves further examination to enable their assessment. Research on the interplay of implicit motives and their appearance in leadership has to first be advanced to yield empirical findings based on which reliable practical implications may be derived that evidently contribute to increasing leadership success in corporate settings.

### Limitations and Directions for Future Research

Although the present study contributes to furthering knowledge in the field of implicit motives and leadership success, it has several limitations and some open questions remain. Directions for future research are emphasized in the light of some of these limitations.

In contrast to studies conducted by De Hoogh et al. (2005), who drew on almost four followers, and Delbecq et al. (2013), who drew on two followers, our study relied on the rating of just one follower. Although we asked the leaders to forward the survey to the follower they worked with closest, they could have chosen the one they liked most (see De Hoogh et al., 2005). Results may thus be distorted by an effect of liking. Additionally, research has found that perceptions of leadership behavior and work-related attitudes are affected by the way followers perceive their leader’s personality and by their own personality traits (Felfe and Schyns, 2010). Therefore, ratings of entire teams may draw a more accurate picture than individual ratings. However, the drawback of relying on teams is that it would be more difficult to find participants. Despite the fact that our sample was in many respects similar in size to other studies on leaders’ implicit motives (Kirkpatrick et al., 2002; De Hoogh et al., 2005; Delbecq et al., 2013), the present sample is admittedly relatively small. Given a larger sample, analyses can be conducted with greater power. Whereas in the present study the power to detect a three-way interaction between *n*Pow, *n*Ach, and *n*Aff on job satisfaction (0.88) and satisfaction with the leader (0.90) exceeded the cut-off value of 0.80 (Cohen, 1962), the power to detect an effect on career success (0.66) or transformational leadership (0.72) was below this value. Therefore, findings urgently have to be replicated in a more comprehensive sample.

The study also failed to show that the examined implicit motives exert an influence on the followers’ performance and attitudes by becoming manifest in transformational leadership. Hence, the way motives affect followers still needs to be examined in more depth. To understand the inner workings of the relation between motives and leadership success, researchers also need to consider the characteristics and attributes related to these motives in addition to the leaders’ behaviors. It is necessary to uncover how these characteristics and attributes translate into followers’ behaviors and attitudes and impact relevant outcomes. For example, motives might manifest themselves in the form of facial expressions, gestures, tone of voice, or other non-verbal features of one’s general behavior. In so doing, we must take into account that these manifestations of the leader’s motives may, in turn, affect their followers’ implicit motives (Schultheiss, 2001). Whether this path of motive arousal helps to explain how the effect of leaders’ motives propagates so as to impact followers, or if and how leaders’ and followers’ motives interact still has to be resolved. Similarly, research has to uncover whether conformity or complementarity of motives is more beneficial for successful leadership, and whether particularly effective combinations of leader-follower motive dispositions exist.

McClelland (1980) reasoned that implicit motives predict operant real-life outcomes that evolve spontaneously (e.g., career success), but not respondent behavior controlled by the environment (e.g., performance tests). Developments in income constitute such an operant outcome. Although career success and motives were both gathered on the part of leaders, the likelihood of common method variance is reduced because motive assessment relies on projective measures. Satisfaction and performance, by contrast, were rated by the followers. Self-reports are appropriate in seizing attitudes (Chan, 2009). Yet they run the risk of constraining a person’s behavior or only eliciting responses based on their pre-selected items. As the validity of implicit motives is confined regarding such respondent measures, a more objective assessment of outcomes may improve future studies. Objective performance data may readily be obtained from key figures, performance evaluations, or organizational records. Objective measures of work-related attitudes are hard to procure. Thus, appropriate operant outcomes need to be considered carefully in future research. As we found the interplay between high *n*Pow, *n*Ach, and *n*Aff adds to the followers’ satisfaction but not to their performance, we assume that different motive combinations will prove differentially effective in predicting various outcomes.

Lastly, our study does not reveal how the manifestation of motives differs depending on whether they are channeled by AI or another motive. Whether substantial differences in a person’s behavior and attributes in fact evolve has to be examined. Given that our results suggest that a leader’s AI directly reduces her or his followers’ job satisfaction, the tendency to inhibit motivational impulses deserves further examination itself, irrespective of its modulating function.

## Conclusion

In sum, our results suggest that successful leadership evolves from the interplay between the leaders’ needs for power, achievement, and affiliation. Although *n*Aff does not seem to foster followers’ performance, this implicit motive is a significant add-on in making followers more satisfied with their jobs and their leaders and in advancing a leader’s career. In this regard, theorizing on implicit motives in leadership needs to be updated.

## Author Contributions

BS, SÖ, and GM substantially contributed to the development of the present study. BS and SÖ compiled the materials and were concerned with data collection. SÖ substantively contributed to the scoring of the Picture Story Exercises. BS, SÖ, and GM were involved in processing, analyzing, and interpreting the data. A draft of the paper developed by BS was discussed among all authors. BS wrote the manuscript and revised it after GM commented on an earlier version of the paper.

## Conflict of Interest Statement

The authors declare that the research was conducted in the absence of any commercial or financial relationships that could be construed as a potential conflict of interest.
